# Supplementary material for: Sequence specificity of an essential nuclear localization sequence in Mcm3
Source: bioRxiv. 2024 Nov 14:2024.11.14.623588. Preprint. [Version 1] doi: 10.1101/2024.11.14.623588 (PMC11601334; doi:10.1101/2024.11.14.623588)
Supplement: 1 [file NIHPP2024.11.14.623588v1-supplement-1.pdf]

bioRxiv preprint doi: https://doi.org/10.1101/2024.11.14.623588; this version posted November 14, 2024. The copyright holder for this preprint (which was not certified by peer review) is the author/funder, who has granted bioRxiv a license to display the preprint in perpetuity. It is made available under aCC-BY 4.0 International license.

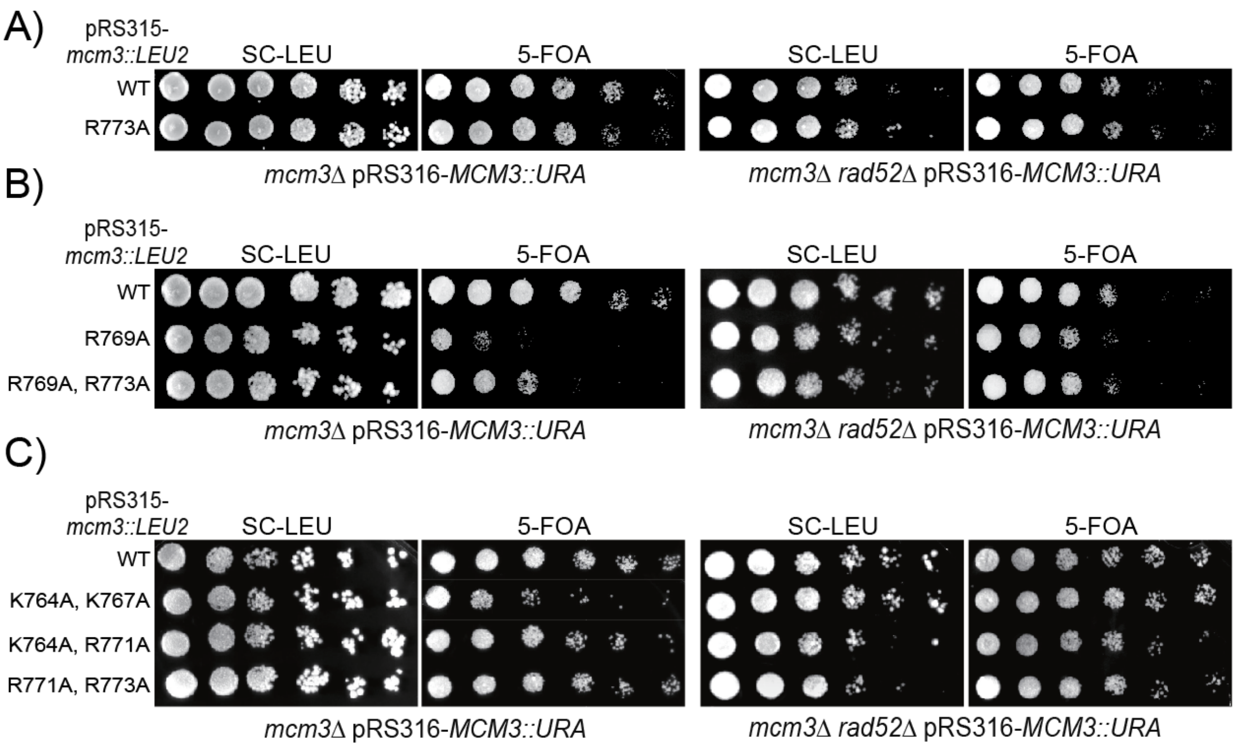

Supplementary Fig 1.

A, B and C) *mcm3* mutants with the indicated mutations were analyzed by plasmid shuffling. Mutants were plated on media where the complementing *MCM3* plasmid was retained (SC-Leu) or lost (5-FOA).

bioRxiv preprint doi: https://doi.org/10.1101/2024.11.14.623588; this version posted November 14, 2024. The copyright holder for this preprint (which was not certified by peer review) is the author/funder, who has granted bioRxiv a license to display the preprint in perpetuity. It is made available under aCC-BY 4.0 International license.

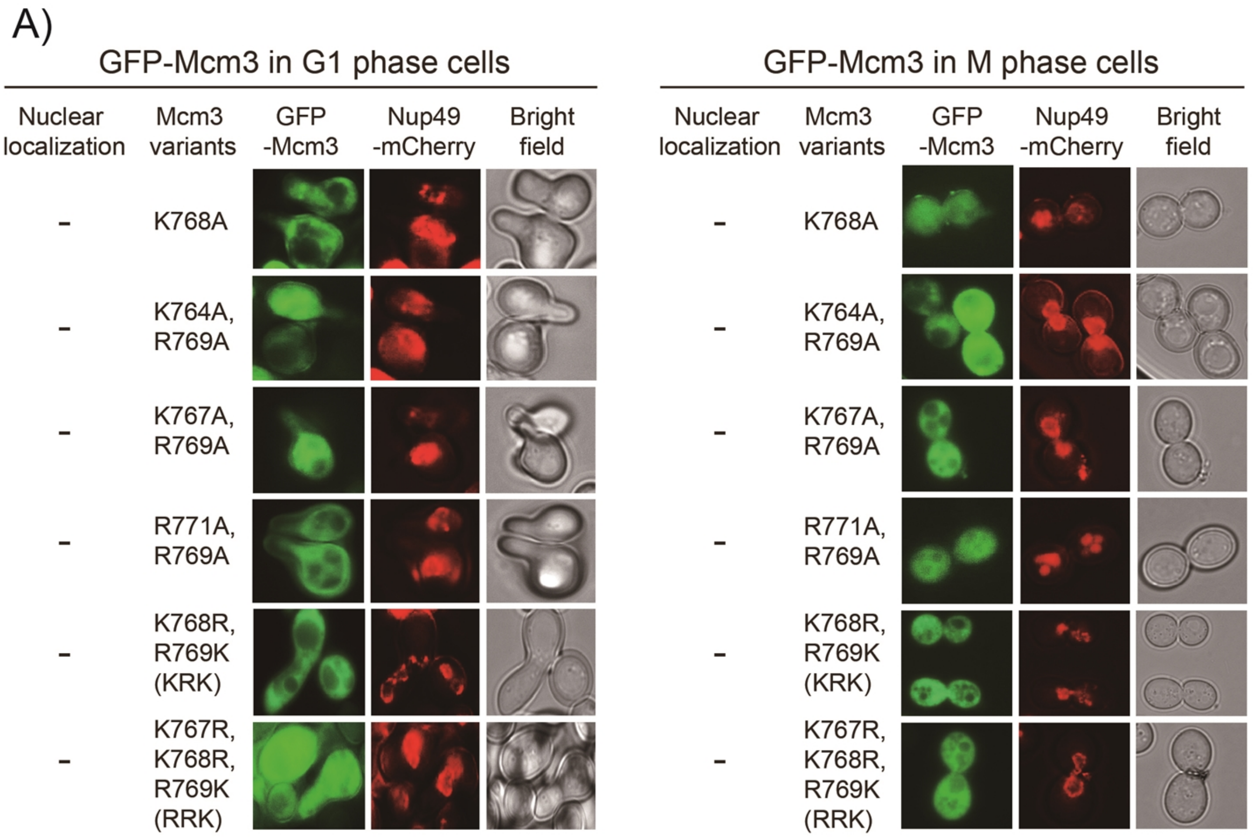

B)

GFP-Mcm3 in G1 phase cells

	WT	K767R, K768R	SV40-NLS	SV40-NLS -allR	K768A	K764A, R769A	K767A, R769A	R771A, R769A	K768R, R769K (KRK)	K767R, K768R, R769K (RRK)
Nuclear	84%	1%	89%	20%	5%	6%	6%	7%	2%	5%
Cytosolic	16%	99%	11%	80%	95%	94%	94%	93%	98%	95%
Total # of Cells	97	114	62	56	60	69	62	57	57	77

GFP-Mcm3 in M phase cells

	WT	K767R, K768R	SV40-NLS	SV40-NLS -allR	K768A	K764A, R769A	K767A, R769A	R771A, R769A	K768R, R769K (KRK)	K767R, K768R, R769K (RRK)
Nuclear	0%	0%	0%	0%	2%	0%	0%	0%	0%	0%
Cytosolic	100%	100%	100%	100%	98%	100%	100%	100%	100%	100%
Total # of Cells	56	52	51	50	53	65	61	57	56	57

Supplementary Fig 2.

A) Nup49-mCherry cells (HZY1575) transformed with pRS315-*GFP-mcm3* plasmids were arrested in G1 or M phase for fluorescence microscopy analysis. Representative images of cells in G1 (left panel) and M (right panel) phases illustrate the localization of GFP-

bioRxiv preprint doi: https://doi.org/10.1101/2024.11.14.623588; this version posted November 14, 2024. The copyright holder for this preprint (which was not certified by peer review) is the author/funder, who has granted bioRxiv a license to display the preprint in perpetuity. It is made available under aCC-BY 4.0 International license.

Mcm3 relative to Nup49-mCherry. B) Quantification data for Fig 3B. The number of cells exhibiting either nuclear or cytosolic localization of the indicated GFP-Mcm3 variants, along with the total number of cells counted are indicated in the table.

bioRxiv preprint doi: https://doi.org/10.1101/2024.11.14.623588; this version posted November 14, 2024. The copyright holder for this preprint (which was not certified by peer review) is the author/funder, who has granted bioRxiv a license to display the preprint in perpetuity. It is made available under aCC-BY 4.0 International license.

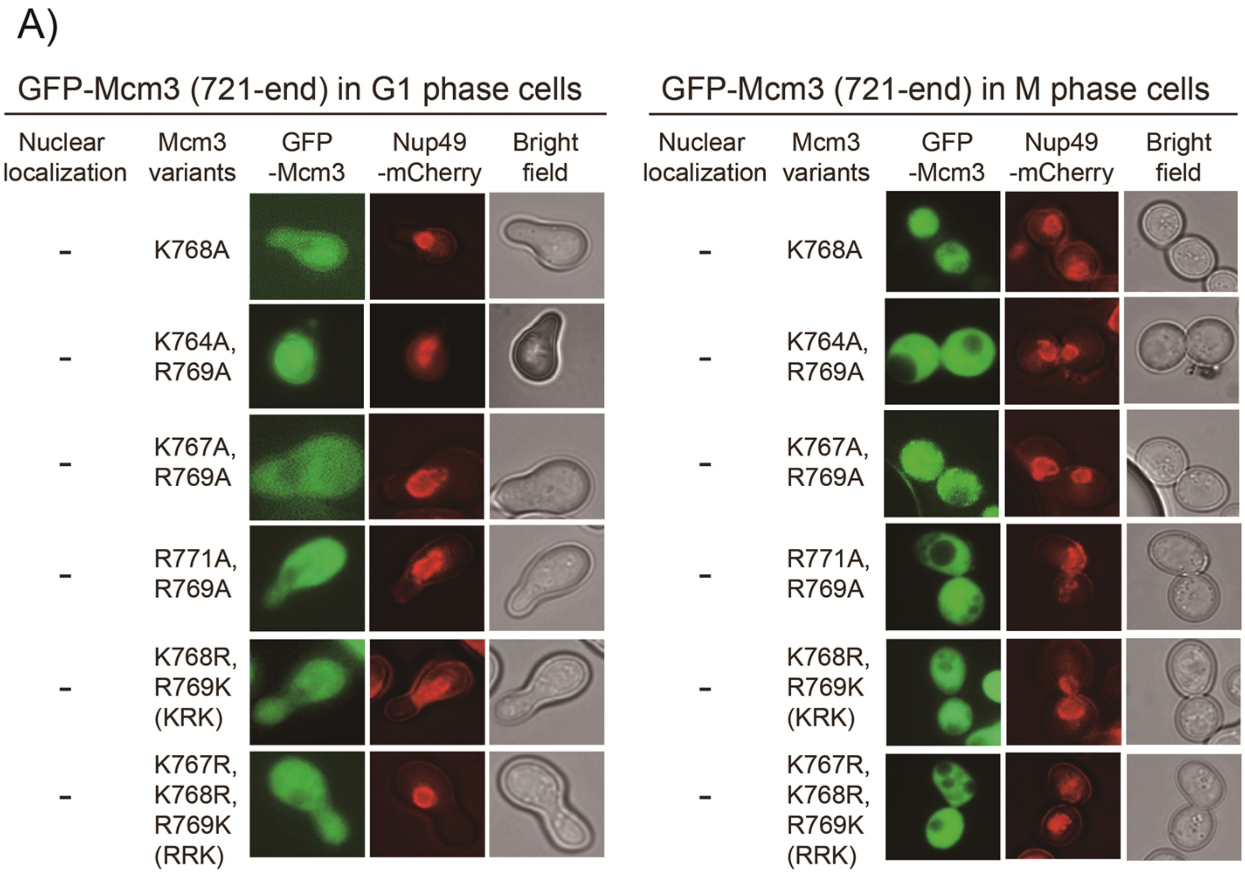

B)

GFP-Mcm3 (721-end) in G1 phase cells

	WT	K767R, K768R	SV40-NLS	SV40-NLS -allR	K768A	K764A, R769A	K767A, R769A	R771A, R769A	K768R, R769K (KRK)	K767R, K768R, R769K (RRK)
Nuclear	67%	6%	89%	1%	2%	3%	6%	5%	6%	4%
Cytosolic	33%	94%	11%	99%	98%	97%	94%	95%	94%	96%
Total # of Cells	79	89	97	114	98	102	85	109	93	100

GFP-Mcm3 (721-end) in M phase cells

	WT	K767R, K768R	SV40-NLS	SV40-NLS -allR	K768A	K764A, R769A	K767A, R769A	R771A, R769A	K768R, R769K (KRK)	K767R, K768R, R769K (RRK)
Nuclear	0%	0%	0%	0%	0%	0%	0%	0%	1%	0%
Cytosolic	100%	100%	100%	100%	100%	100%	100%	100%	99%	100%
Total # of Cells	53	73	66	54	89	100	73	119	81	97

Supplementary Fig 3.

A) Nup49-mCherry cells (HZY1575) transformed with pRS315-*GFP-mcm3-721-end* plasmids were arrested in G1 or M phase for fluorescence microscopy analysis.

bioRxiv preprint doi: https://doi.org/10.1101/2024.11.14.623588; this version posted November 14, 2024. The copyright holder for this preprint (which was not certified by peer review) is the author/funder, who has granted bioRxiv a license to display the preprint in perpetuity. It is made available under aCC-BY 4.0 International license.

Representative images of cells in G1 (left panel) and M (right panel) phases illustrate the localization of GFP-Mcm3 (721-end) relative to Nup49-mCherry. B) Quantification data for Fig 4B. The number of cells exhibiting either nuclear or cytosolic localization of the indicated GFP-Mcm3 (721-end) variants, along with the total number of cells counted are indicated in the table.

bioRxiv preprint doi: https://doi.org/10.1101/2024.11.14.623588; this version posted November 14, 2024. The copyright holder for this preprint (which was not certified by peer review) is the author/funder, who has granted bioRxiv a license to display the preprint in perpetuity. It is made available under aCC-BY 4.0 International license.

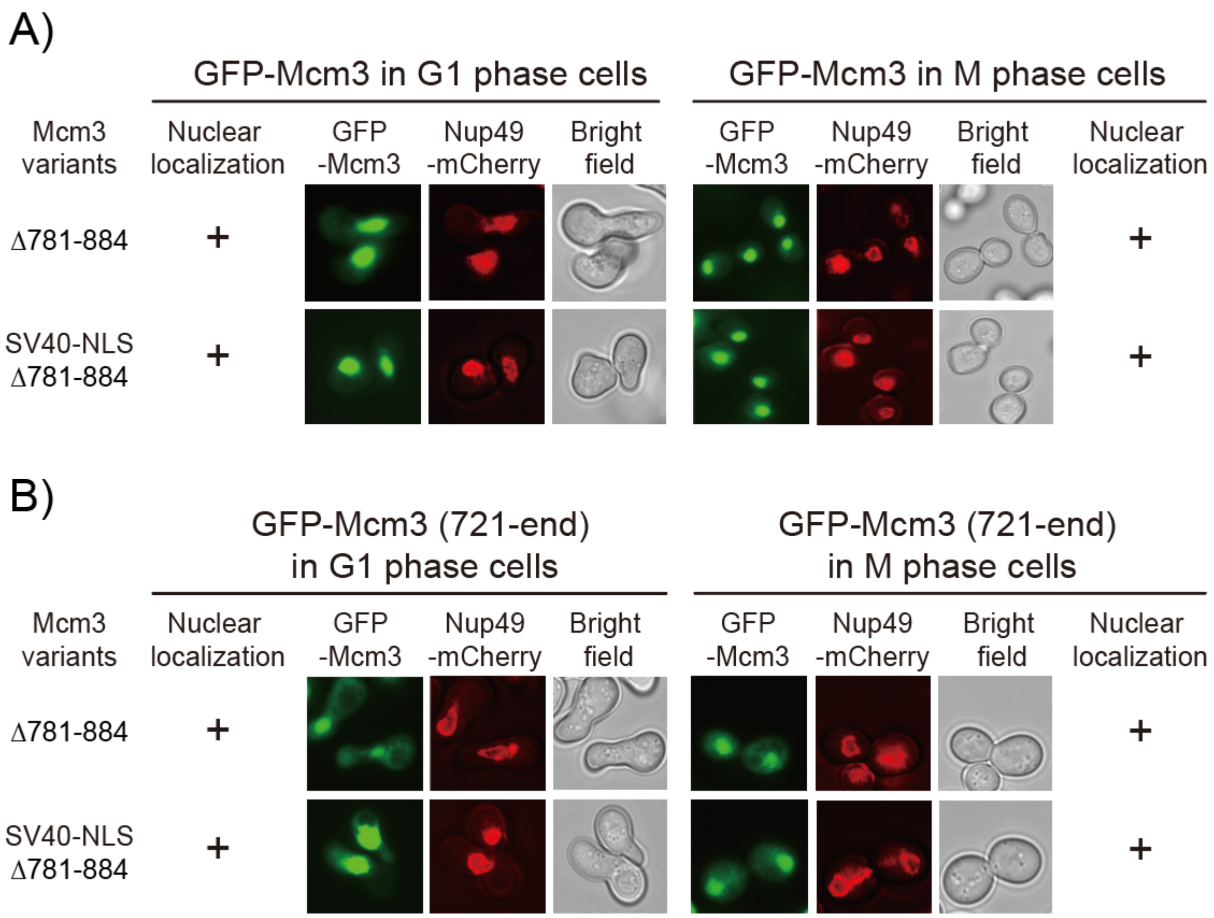

Supplementary Fig 4.

A and B) Nup49-mCherry cells (HZY1575) transformed with pRS315-*GFP-mcm3* or pRS315-*GFP-mcm3-721-end* plasmids were arrested in G1 or M phase for fluorescence microscopy analysis. Representative images of cells in G1 (left panel) and M (right panel) phases illustrate the localization of GFP-Mcm3 (Fig S4A) or GFP-Mcm3 (721-end) (Fig S4B) relative to Nup49-mCherry.

bioRxiv preprint doi: https://doi.org/10.1101/2024.11.14.623588; this version posted November 14, 2024. The copyright holder for this preprint (which was not certified by peer review) is the author/funder, who has granted bioRxiv a license to display the preprint in perpetuity. It is made available under aCC-BY 4.0 International license.

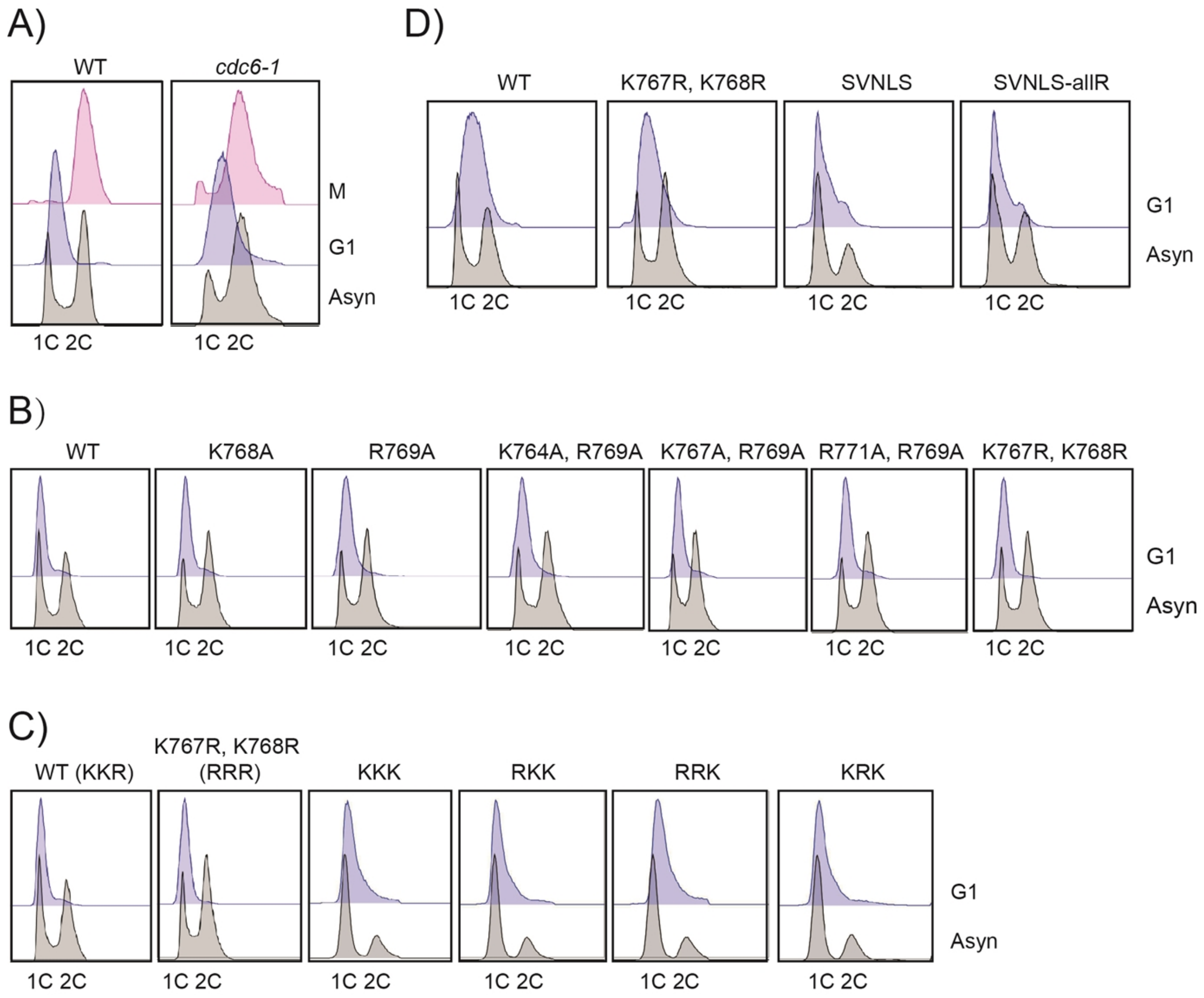

Supplementary Fig 5. A) Fluorescence-Activated Cell Sorting (FACS) analysis of wild-type (HZY4011) and *cdc6-1* (HZY1366) cells following G1 and M phase arrest for Fig 5B. B) FACS analysis of *mcm3* mutants following G1 arrest for Figs 5C, S6D to S6F. C) FACS analysis of *mcm3* mutants following G1 arrest for Figs 5D and S6G. D) FACS analysis of *mcm3* mutants following G1 arrest for Figs 5D and S6H.

bioRxiv preprint doi: https://doi.org/10.1101/2024.11.14.623588; this version posted November 14, 2024. The copyright holder for this preprint (which was not certified by peer review) is the author/funder, who has granted bioRxiv a license to display the preprint in perpetuity. It is made available under aCC-BY 4.0 International license.

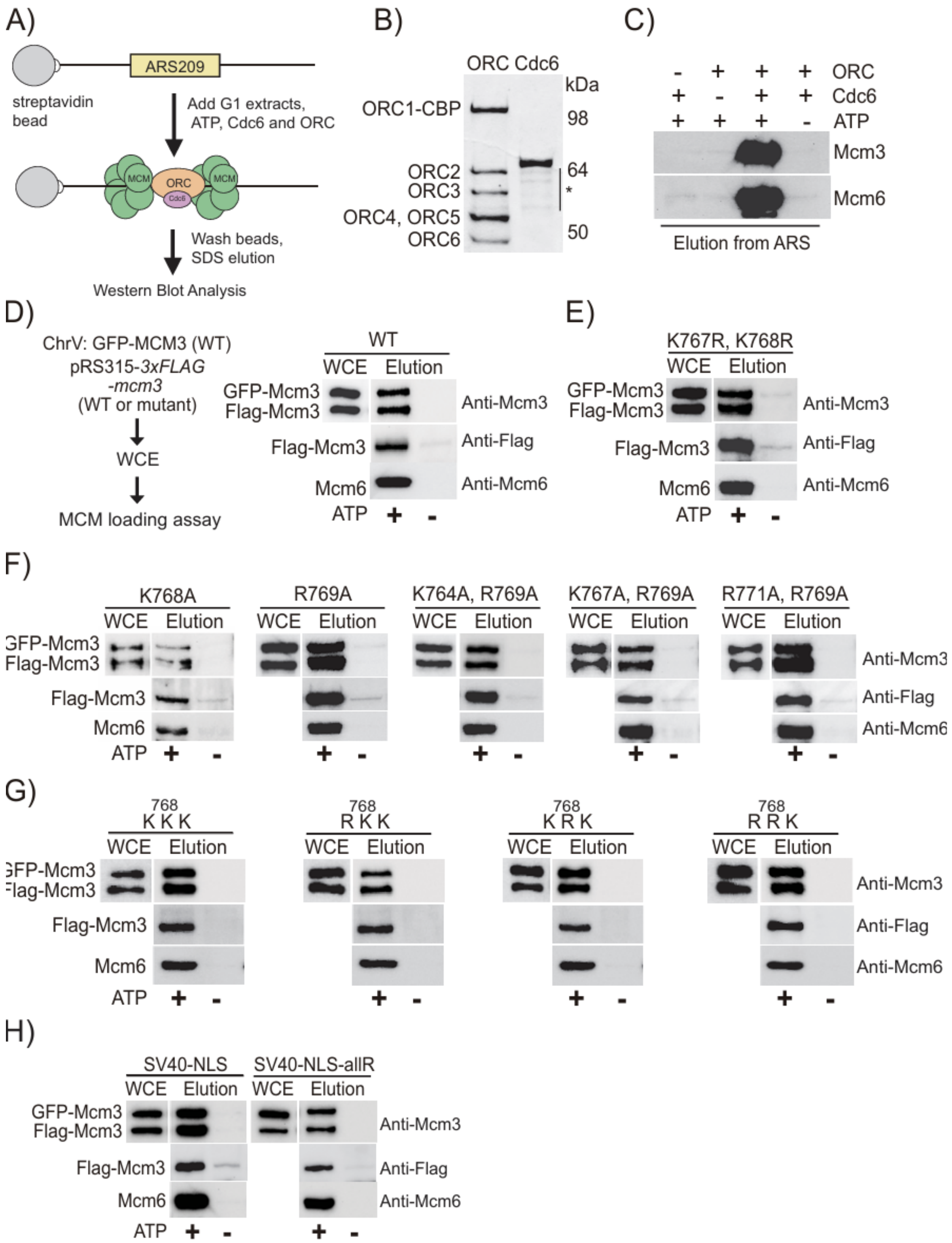

bioRxiv preprint doi: https://doi.org/10.1101/2024.11.14.623588; this version posted November 14, 2024. The copyright holder for this preprint (which was not certified by peer review) is the author/funder, who has granted bioRxiv a license to display the preprint in perpetuity. It is made available under aCC-BY 4.0 International license.

Supplementary Fig 6. A) A workflow for loading MCM on ARS-containing DNA in vitro. B) Purified ORC and Cdc6 proteins were analyzed by SDS–PAGE and stained with Coomassie blue dye. * Indicates partial degradation of the proteins. C) G1 phase whole cell extracts (WCE) of wild-type (HZY4011) cells were subjected to *in vitro* MCM loading assay under different conditions: with or without ATP, Cdc6, or ORC. ARS DNA-bound materials were eluted by SDS and analyzed by Western blotting with anti-Mcm3 and anti-Mcm6 antibodies. D to H) GFP-Mcm3 cells (HZY3037) transformed with *pRS315-3xFLAG-mcm3* plasmids were arrested in the G1 phase, and the corresponding whole cell extracts (WCE) were subjected to in vitro MCM loading assay. ARS DNA-bound materials were eluted by SDS and analyzed by Western blotting with anti-Mcm3 and anti-Flag antibodies.

bioRxiv preprint doi: https://doi.org/10.1101/2024.11.14.623588; this version posted November 14, 2024. The copyright holder for this preprint (which was not certified by peer review) is the author/funder, who has granted bioRxiv a license to display the preprint in perpetuity. It is made available under aCC-BY 4.0 International license.

A)

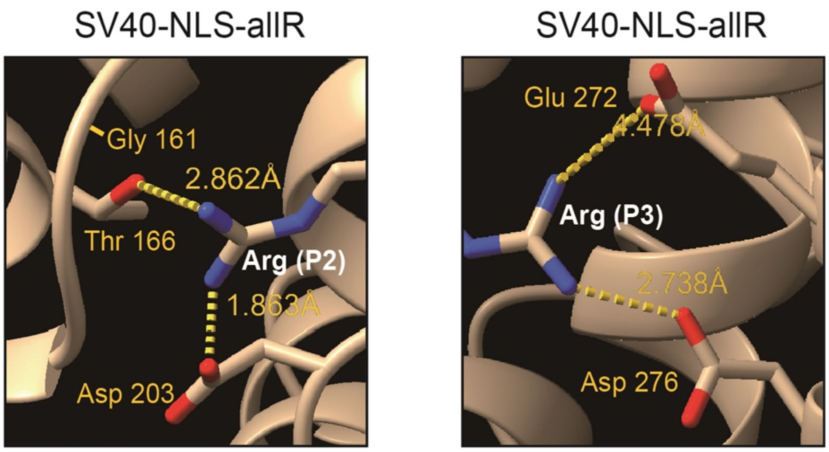

B)

Mcm3 mutant	P2		P3	
	Thr166	Asp203	Asp276	Glu272
SV40-NLS	2.8Å	2.6Å	4.5Å	4.4Å
SV40-NLS (AlphaFold)	2.777Å	2.674Å	4.198Å	4.736Å
SV40-NLS-allR	2.862Å	1.863Å	2.738Å	4.478Å
WT	2.785Å	2.621Å	3.467Å	4.309Å
K764A	2.879Å	2.635Å	3.143Å	4.422Å
K767A	2.84Å	2.653Å	3.207Å	4.891Å
K764A, K767A	2.797Å	2.636Å	4.141Å	4.534Å
K767R	2.754Å	2.642Å	3.974Å	4.612Å
K768A			4.037Å	4.669Å
K768R				
K767R, K768R				
K767R, K768R, R769K				
K767R, R769K	2.852Å	2.663Å	4.299Å	4.802Å
R769K	2.842Å	2.647Å	4.214Å	4.867Å
K768R, R769K				
R769A				
R771A	2.657Å	2.835Å	3.049Å	4.62Å
K764A, R771A	2.625Å	2.761Å	4.104Å	4.643Å
K764A, R769A				
K767A, R769A	2.821Å	2.611Å		
R771A, R769A				

Supplementary Fig 7. A) AlphaFold predicted interaction between the basic residue at P2 (left panel) or P3 (right panel) in Mcm3$^{SV40-NLS-allR}$ (^{764}KSPRRRRRV^{772}) and Kap60. B) The distances between residues at P2 or P3 within Mcm3's NLS and key residues in Kap60 were modeled by AlphaFold. Orange indicates that, according to AlphaFold 3 modeling, no specific contact was detected that resembled the published structure of SV40-NLS/karyopherin alpha complex.

bioRxiv preprint doi: https://doi.org/10.1101/2024.11.14.623588; this version posted November 14, 2024. The copyright holder for this preprint (which was not certified by peer review) is the author/funder, who has granted bioRxiv a license to display the preprint in perpetuity. It is made available under aCC-BY 4.0 International license.

Supplementary Table 1. Yeast strains used.

HZY1077	W303 MATa, *ade2-1*, *can1-100*, *his3-1115*, *leu2-3,112*, *trp1-1*, *ura3-1*, *RAD5+*	This study
HZY1078	W303 MATalpha, *ade2-1*, *can1-100*, *his3-1115*, *leu2-3,112*, *trp1-1*, *ura3-1*, *RAD5+*	This study
HZY1079	W303 Diploid of HZY1077 and HZY1078	This study
HZY232	W303 Diploid, *mcm3Δ::NAT* in HZY1079	This study
HZY234	W303 MATa, *mcm3Δ::NAT*, pRS316-*MCM3,* derived from HZY232	This study
HZY3732	W303 MAT alpha, *rad52Δ::G418*	This study
HZY3734	W303 Diploid, *rad52Δ::G418/RAD52*, *mcm3Δ::NAT/MCM3*, pRS316-*MCM3,* derived from HZY3732xHZY234	This study
HZY2218	W303 MATa, *mcm3Δ::NAT*, *rad52Δ::G418, pRS316-MCM3,* derived from HZY3734	This study
HZY4011	W303 MATa, *bar1Δ::hyg* in HZY1077	This study
HZY1366	W303 MATa, *cdc6-1 (G260D)::LEU2*, *bar1D::URA*, *Mcm4-TAF::HIS*	This study
HZY3037	MATa, *GFP-Mcm3::HIS*, *bar1D::Ura3*	This study
yJF2966	MATa, *TRP1*::GAL1-10-*ORC5-ORC6*, *HIS3*::GAL1-10-*ORC3-ORC4*, *URA3::GAL1-10-CBP-TEV-ORC1-ORC2*, *ade2-1, ura3-1, his3-11,15 trp1-1 leu2-3,112 can1-100, bar1::hyg, pep4::kanMX6*	Frigola, *et al.* 2013
HZY549	S288C MATa, *ura3-52, leu2-1, trp1-63, his3-200, lys2-Bgl, hom3-10, ade2-1, ade8, sml1Δ::TRP, arg4Δ*	This study
HZY1128	MATa, *bar1Δ::HIS, sml1Δ::TRP,* derived from HZY549	This study
HZY1506	MATa, *Mcm6-TAF::G418* in HZY1128	This study
HZY329	MATalpha, *NUP49-mCherry::hphNT1, leu-,* derived from diploid (*arg4Δ/ARG4, sml1Δ::TRP1/SML1,*	This study

bioRxiv preprint doi: https://doi.org/10.1101/2024.11.14.623588; this version posted November 14, 2024. The copyright holder for this preprint (which was not certified by peer review) is the author/funder, who has granted bioRxiv a license to display the preprint in perpetuity. It is made available under aCC-BY 4.0 International license.

	trp1Δ63/trp1Δ63, ura3-52/ura3Δ0, leu2Δ1/leu2Δ0, his3Δ200/his3Δ200 lys2ΔBgl/LYS2, hom3-10, ade2Δ1/ADE2, ade8/ADE8, can1/CAN1, cyh2-Q38K, YFR016C::Pmfa1-Leu2)	
HZY1575	MATa, *NUP49-mCherry::hphNT1/hygro, bar1Δ::HIS sml1D::TRP1*, derived from HZY329xHZY1506	This study

bioRxiv preprint doi: https://doi.org/10.1101/2024.11.14.623588; this version posted November 14, 2024. The copyright holder for this preprint (which was not certified by peer review) is the author/funder, who has granted bioRxiv a license to display the preprint in perpetuity. It is made available under aCC-BY 4.0 International license.

Supplementary Table 2. Plasmids used

HZE1997	pRS316-*MCM3*	This study
HZE3004	pRS315-*3xFLAG-MCM3*	This study
HZE3018	pRS315-*3xFLAG-mcm3 (Δ742-758)*	This study
HZE3111	pRS315-*3xFLAG-mcm3 (Δ771-780)*	This study
HZE3112	pRS315-*3xFLAG-mcm3 (Δ781-790)*	This study
HZE3366	pRS315-*3xFLAG-mcm3 (Δ791-885)*	This study
HZE2019	pRS315-*MCM3*	This study
HZE3000	pRS315-*mcm3- K764A:HIS3*	This study
HZE3001	pRS315-*mcm3- S765A:HIS3*	This study
HZE3002	pRS315-*mcm3- P766A:HIS3*	This study
HZE3052	pRS315-*3xFLAG-mcm3-K767A*	This study
HZE3053	pRS315-*3xFLAG-mcm3-K768A:HIS3*	This study
HZE3054	pRS315-*3xFLAG-mcm3-R769A*	This study
HZE3076	pRS315-*3xFLAG-mcm3-Q770A*	This study
HZE3077	pRS315-*3xFLAG-mcm3-R771A*	This study
HZE3413	pRS315-*3xFLAG-mcm3-R773A*	This study
HZY3081	pRS315-*3xFLAG-mcm3-2KR*	This study
HZE3221	pRS315-*3xFLAG-mcm3-K767A, R769A*	This study
HZE3412	pRS315-*3xFLAG-mcm3-K764A, R769A*	This study
HZE3401	pRS315-*3xFLAG-mcm3-R769A, R771A*	This study
HZE3372	pRS315-*3xFLAG-mcm3-R769K (KKK)*	This study
HZE3373	pRS315-*3xFLAG-mcm3-K767R, R769K (RKK)*	This study
HZE3374	pRS315-*3xFLAG-mcm3-K768R, R769K (KRK)*	This study
HZE3375	pRS315-*3xFLAG-mcm3-K767R, K768R, R769K (RRK)*	This study
HZE1134	pGEX-6P1-*CDC6*	This study
HZE3416	pRS315-*3xFLAG-mcm3-SV40-NLS (R769K, Q770R, R771K)*	This study

bioRxiv preprint doi: https://doi.org/10.1101/2024.11.14.623588; this version posted November 14, 2024. The copyright holder for this preprint (which was not certified by peer review) is the author/funder, who has granted bioRxiv a license to display the preprint in perpetuity. It is made available under aCC-BY 4.0 International license.

HZE3417	pRS315-*3xFLAG-mcm3-SV40-NLS-allR (K767R, K768R, Q770R)*	This study
HZE2187	pRS315-*GFP-MCM3*	This study
HZE2190	pRS315-*GFP-mcm3-2KR*	This study
HZE2315	pRS315-*GFP-mcm3-721-end*	This study
HZE2318	pRS315-*GFP-mcm3-721-end-2KR (K767R, K768R)*	This study
HZE3421	pRS315-*GFP-mcm3-721-end-SV40-NLS*	This study
HZE3422	pRS315-*GFP-mcm3-721-end-SV40-NLS-allR*	This study
HZE3446	pRS315-*GFP-mcm3-R769A, R771A*	This study
HZE3447	pRS315-*GFP-mcm3-AKA (K767A, K768 and R769A)*	This study
HZE3448	pRS315-GFP-*mcm3-KRK (K768R and R769K)*	This study
HZE3449	pRS315-GFP-*mcm3-RRK (K767R, K768R and R769K)*	This study
HZE3450	pRS315-GFP-*mcm3-721-end-RRK (K767R, K768R and R769K)*	This study
HZE3451	pRS315-GFP-*mcm3-721-end-KRK (K768R and R769K)*	This study
HZE3452	pRS315-GFP-*mcm3-721-end (K764A and R769A)*	This study
HZE3453	pRS315-GFP-*mcm3 (K768A)::HIS3*	This study
HZE3454	pRS315-GFP-*mcm3 (K764A, R769A)*	This study
HZE3456	pRS315-GFP-*mcm3-721-end (R771A and R769A)*	This study
HZE3457	pRS315-GFP-*mcm3-SV40-NLS-allR*	This study
HZE3458	pRS315-GFP-*mcm3-721-end-AKA (K767A, K768 and R769A)*	This study
HZE3459	pRS315-GFP-*mcm3-SV40-NLS*	This study
HZE3461	pRS315-GFP-*mcm3-721-end-K768A*	This study
HZE3468	pRS315-GFP-*mcm3 (Δ781-884)*	This study
HZE3470	pRS315-GFP-*mcm3-SV40 (Δ781-884)*	This study
HZE3471	pRS315-GFP-*mcm3-721-end (Δ781-884)*	This study
HZE3473	pRS315-GFP-*mcm3-SV40NLS-721-end (Δ781-884)*	This study

bioRxiv preprint doi: https://doi.org/10.1101/2024.11.14.623588; this version posted November 14, 2024. The copyright holder for this preprint (which was not certified by peer review) is the author/funder, who has granted bioRxiv a license to display the preprint in perpetuity. It is made available under aCC-BY 4.0 International license.

HZE3047	pRS316-*ΔCEN6*	This study
